# Comparison of Photoselective Vaporization versus Holmium Laser Enucleation for Treatment of Benign Prostate Hyperplasia in a Small Prostate Volume

**DOI:** 10.1371/journal.pone.0156133

**Published:** 2016-05-26

**Authors:** Kang Sup Kim, Jin Bong Choi, Woong Jin Bae, Su Jin Kim, Hyuk Jin Cho, Sung-Hoo Hong, Ji Youl Lee, Sang Hoon Kim, Hyun Woo Kim, Su Yeon Cho, Sae Woong Kim

**Affiliations:** 1 Department of Urology, College of Medicine, The Catholic University of Korea, Seoul St Mary’s hospital, Seoul, Korea; 2 Department of Urology, College of Medicine, The Catholic University of Korea, St Paul’s hospital, Seoul, Korea; University of Michigan, UNITED STATES

## Abstract

**Objective:**

Photoselective vaporization of the prostate (PVP) using GreenLight and Holmium laser enucleation of the prostate (HoLEP) is an important surgical technique for management of benign prostate hyperplasia (BPH). We aimed to compare the effectiveness and safety of PVP using a 120 W GreenLight laser with HoLEP in a small prostate volume.

**Methods:**

Patients who underwent PVP or HoLEP surgery for BPH at our institutions were reviewed from May 2009 to December 2014 in this retrospective study. Among them, patients with prostate volumes < 40 mL based on preoperative trans-rectal ultrasonography were included in this study. Peri-operative and post-operative parameters—such as International Prostate Symptom Score (IPSS), quality of life (QoL), maximum urinary flow rate (Qmax), post-void residual urine volume (PVR), and complications—were compared between the groups.

**Results:**

PVP was performed in 176 patients and HoLEP in162 patients. Preoperative demographic data were similar in both groups, with the exception of PVR. Operative time and catheter duration did not show significant difference. Significant improvements compared to preoperative values were verified at the postoperative evaluation in both groups in terms of IPSS, QoL, Qmax, and PVR. Comparison of the postoperative parameters between the PVP and HoLEP groups demonstrated no significant difference, with the exception of IPSS voiding subscore at 1 month postoperatively (5.9 vs. 3.8, P< 0.001). There was no significant difference in postoperative complications between the two groups.

**Conclusion:**

Our data suggest that PVP and HoLEP are efficient and safe surgical treatment options for patients with small prostate volume.

## Introduction

Benign prostatic hyperplasia (BPH) is a common condition in aging males and is the most common cause of lower urinary tract symptoms (LUTS) [1]. Therapeutic options for BPH have increased during the last decade, and range from medical treatment to surgery. Although transurethral resection of the prostate (TURP) is regarded as the surgical gold standard for treating bladder outlet obstruction (BOO) secondary to BPH, various lasers and advanced techniques have been introduced as alternatives to TURP because of their low morbidity and high efficiency. Two of the most common are photoselective vaporization of the prostate (PVP) using 120 W GreenLight laser and holmium:YAG laser enucleation of the prostate (HoLEP).

The association of prostate volume and LUTS in patients with BPH remains controversial despite numerous studies. Some studies reported a poor correlation between prostate volume and voiding symptoms [2, 3]. Although surgical treatment of LUTS secondary to small prostate volume is controversial, it is regarded as an alternative treatment for patients refractory to medical therapy. Reich et al. reported the morbidities and early outcomes of TURP for a small prostate volume. [4] Low rates of transfusion, TUR syndrome, and mortality, improved urinary peak flow rate (Qmax) and decreased post-void residual urine (PVR) were reported in this prospective multicenter study. PVP was comparable with TURP in terms of voiding symptoms and complications in randomized studies [5, 6]. In a study comparing TURP with HoLEP with matched prostate volumes, HoLEP is an effective treatment modality in a small prostate volume [7]. Kuntz et al. evaluated the efficacy of HoLEP in small, medium-size, and large prostates [8]. The authors concluded that prostate volume does not affect performance of HoLEP, and HoLEP is equally effective in small, medium, and large prostate volumes. Therefore, PVP and HoLEP have been used in patients with small-to-medium-sized BPH causing BOO according to international guidelines. However, few studies have directly compared PVP and HoLEP in patients with a small prostate volume [9]. Herein, the present study analyzed the efficacy and safety of the PVP using 120W GreenLight laser versus HoLEP in patients with LUTS due to a small prostate volume (< 40 mL).

## Materials and Methods

### Study population

After approval by the Institutional Review Board at the Catholic University of Korea, Seoul St. Mary’s Hospital prospectively collected GreenLight and HoLEP data were reviewed for patients who underwent operations for BOO secondary to a small prostate volume at two Saint’s Mary Hospital between May 2009 and December 2014. All patients’ records/information were anonymized and de-identified prior to analysis. A complete medical history, digital rectal examination, International Prostate Symptom Score (IPSS) questionnaire, urinalysis, serum creatinine examination, determination of serum prostate-specific antigen (PSA), Qmax, PVR, transrectal ultrasonography (TRUS), and urodynamic study were executed in all patients before surgical treatment.

Inclusion criteria for the study were moderate-to-severe LUTS (IPSS score > 7, Qmax < 15mL/s, PVR > 100 mL), recurrent urinary retention, persistent gross hematuria from the prostate, recurrent urinary tract infection, and bladder stones. Patients with prostate or bladder carcinoma, neurogenic bladder dysfunction, urethral stricture, or previous pelvic surgery were excluded. If patients had a PSA level > 4.0 ng/mL, DRE abnormalities, or hypoechogenic lesion in TRUS, a TRUS-guided prostate biopsy was performed to exclude a malignancy. Prostate volume was measured using TRUS and calculated using a conventional formula (length * width* height * π/6). All patients in the present study had a prostate volume < 40 mL.

### Surgical technique

Four surgeons performed a standardized PVP and HoLEP technique at the two hospitals. PVP and HoLEP were performed according to steps described previously [10, 11]. PVP was implemented using a GreenLight 120 W HPS system (American Medical System Inc., Minnetonka, MN, USA). A laser fiber was inserted through the working channel of a continuous double flow 22 Fr resectoscope with normal saline irrigation. Vaporization using a side-to-side sweeping technique was initiated at the bladder neck area and moved to the level of the verumontanum. Power setting used to vaporize the bladder neck was 60–80 W and that for the lateral lobe was 80–120 W. Prostate tissue causing BOO was eliminated as much as possible until a TURP-like cavity was formed. A 20 Fr. three-way Foley catheter was placed after the operation, and irrigation using normal saline was begun in the operating room. Urethral catheters were usually removed on the first day postoperatively after the urine color became clear. Patients were discharged home once they could void well. The urethral catheters of patients unable to urinate were replaced before discharge. Patients attempted to void after removal of the urethral catheter at an outpatient clinic follow up. HoLEP was performed using a 100 W holmium:YAG laser (VersaPulse PowerSuite, Lumenis Surgical, San Jose, CA, USA) with a 550nm end-firing fiber (SlimLine, Lumenis). A 26Fr. continuous-flow resectoscope with saline irrigation was used; the laser settings were 2.5 J and 40Hz. After enucleation was performed using a two- or three-lobe technique and bleeding control achieved, enucleated adenoma tissue was removed from the urinary bladder using a mechanical tissue morcellator (Versa-Cut, Lumenis) via an indirect nephroscope. Subsequent patient care was similar to that applied post-PVP.

### Follow up

Changes in subjective (IPSS and QoL) and objective (Qmax and PVR) parameters between the PVP and HoLEP groups were evaluated and compared at 1, 3, 6, and 12 months after the operation. Perioperative (operative time, catheterization time) parameters and early and late postoperative complications—such as acute urinary retention, gross hematuria, acute urinary tract infection, urethral stricture, and bladder neck contracture (BNC)—were also compared between the two groups.

### Statistical analysis

All statistical analyses were conducted using the SPSS software (SPSS, Inc., Chicago, IL, USA). Continuous variables are presented as medians (range) or means and standard deviation and qualitative variables are expressed as frequencies with percentages. Results were compared between two groups using Student’s *t*-test and Mann-Whitney U test for continuous variables and chi-squared test and Fisher’s exact test for qualitative variables. A P-value < 0.05 was considered significant.

## Results

A total of 338 patients (PVP group: 176, HoLEP: 162) with small prostate volume accorded to the inclusion criteria were included in the study. The demographic and disease characteristics of the patients are presented in Table 1. The mean age of the patients in the PVP and HoLEP groups was 70.7 and 69.5 years, respectively (P = 0.093). The mean prostate volume was similar in the two groups (30.2 vs. 29.2 mL, P = 0.091). There was no significant difference between the two groups with respect to preoperative PSA (2.0 vs. 1.9 mg/mL, P = 0.300), total IPSS score (20.4 vs. 21.5, P = 0.241), IPSS QoL score (4.1 vs. 4.2, P = 0.640), Qmax (8.7 vs. 9.3 mL/s, P = 0.174). However, PVR was larger in the PVP group (133 vs. 86.8 mL, P < 0.001).

**Table 1:** Demographic characteristics.

	PVP (n = 176)	HoLEP (n = 162)	P-value
**Age**	70.7 ± 8.1	69.5 ± 7.4	0.093
**Preop PSA (ng/mL)**	2.0 ± 1.8	1.9 ± 1.9	0.300
**Prostate size by TRUS (mL)**	30.2 ± 6.1	29.2 ± 6.7	0.091
**IPSS total score**	20.4 ± 7.8	21.5 ± 8.5	0.241
**IPSS voiding score**	12.1 ± 5.2	13.2 ± 5.4	0.057
**IPSS storage score**	8.3 ± 3.8	8.3 ± 4.3	0.940
**IPSS QoL**	4.1 ± 2.2	4.2 ± 1.1	0.640
**Qmax (mL/s)**	8.7 ± 4.9	9.3 ± 4.3	0.174
**PVR (mL)**	133.0± 115.7	86.8 ± 116.3	<0.001

Values are means ± standard deviation. PVP, photoselective vaporization of prostate; HoLEP holmium laser enucleation of the prostate; PSA, prostate-specific antigen; IPSS, International Prostate Symptoms Score; QoL, quality of life; Qmax, urinary peak flow rate; PVR, post-voiding residual urine.

Table 2 summarizes the perioperative parameters and complications after PVP and HoLEP. There was no significant difference operative time, indwelling catheter duration between the groups. However, compared with the HoLEP group, patients in the PVP group had higher total energy usage (91.1 vs. 83.9 KJ, P = 0.041). The incidence of complications was not significantly different between the PVP and HoLEP groups during the follow-up (6.8% vs. 3.7%, P = 0.635). Urethral stricture occurred in 2.3% of patients after PVP and in 2.5% after HoLEP. BNC was observed in six cases (3.4%) in the PVP group and in two cases (1.2%) in the HoLEP group.

**Table 2:** Perioperative data and adverse events in patients with small prostate volume undergoing PVP or HoLEP.

	PVP (n = 176)	HoLEP (n = 162)	P value
**Operative time (min)**	51.4 ± 20.1	47.6 ± 26.8	0.087
**Total energy used (KJ)**	94.1 ± 76.7	83.9 ± 36.7	0.041
**Enucleation time (min)**		38.2 ± 27.9	
**Morcellation time (min)**		9.1 ± 7.1	
**Enucleated tissue weight (gm)**		12.0 ± 7.0	
**Indwelling catheter time (days)**	1.8 ± 2.0	2.1 ± 1.5	0.138
**Total complications (%)**	12 (6.8%)	6 (3.7%)	0.635
**Gross hematuria**	1 (0.6%)	0 (0%)	
**Febrile UTI**	1 (0.6%)	1 (0.6%)	
**Urethral stricture**	4 (2.3%)	4 (2.5%)	
**BNC**	6 (3.4%)	1 (1.2%)	

Values are means ± standard deviation or frequencies with percentages. PVP, photoselective vaporization of prostate; HoLEP holmium laser enucleation of the prostate; UTI, urinary tract infection; BNC, bladder neck contracture

**Fig 1:**
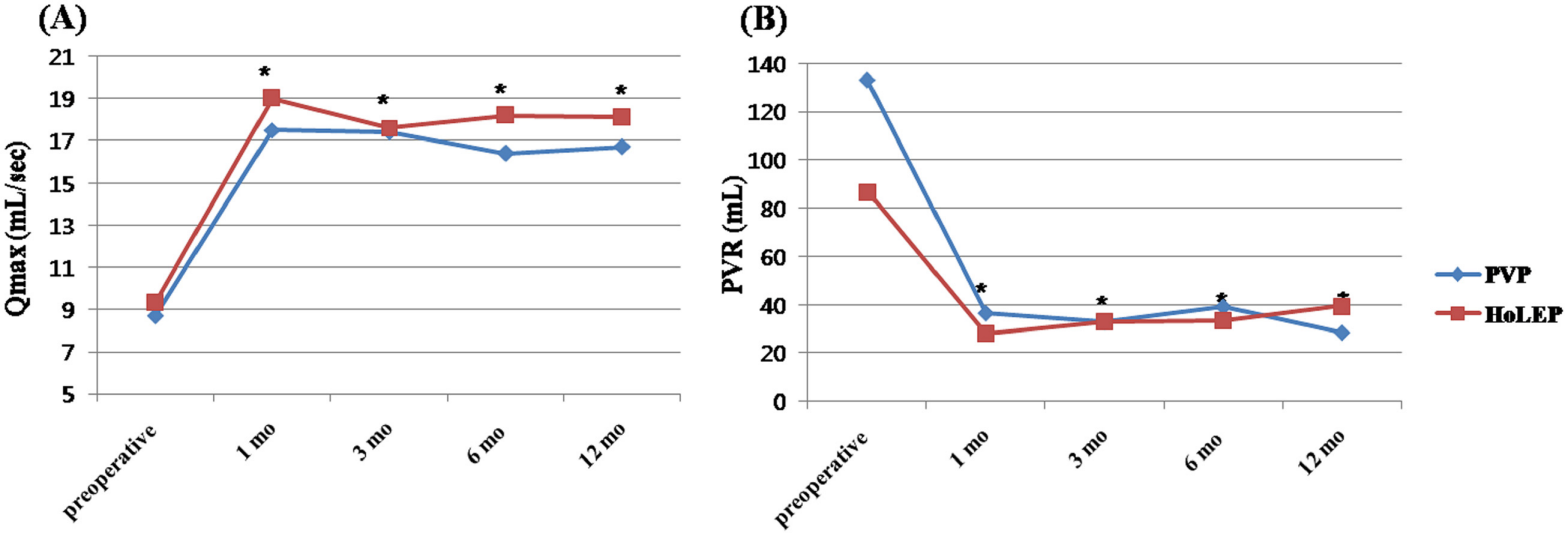
Changes in Qmax (A) and PVR (B) in the PVP and HoLEP groups. Qmax, urinary peak flow rate; PVR, post-voiding residual urine; PVP, photoselective vaporization of the prostate; HoLEP, holmium laser enucleation of the prostate. * P < 0.05; compared with preoperative parameters.

Subjective follow-up data are shown in Table 3. At the 12-month follow-up, compared with preoperative data, significant ameliorations of the total IPSS score, voiding IPSS subscore, storage IPSS subscore, and QoL were observed after the operation (P <0.05). However, the differences between both groups were not significant, with the exception of the voiding IPSS subscore at 1 month postoperatively (5.9 vs. 3.8, P < 0.001). Objective follow up data, including Qmax and PVR, are shown in Fig 1. Qmax and PVR improved significantly compared to baseline beginning1month after surgery in both the PVP and HoLEP groups; this improvement was sustained throughout the 12 months of follow up.

**Table 3:** Follow-up data of up to 12 months after PVP or HoLEP.

	Preoperative	Postoperative			
		1 month	3 months	6 months	12 months
**IPSS total**					
**PVP**	20.4 ± 7.8	11.6 ± 7.0	9.4 ± 4.9	9.6 ± 6.1	9.7 ± 5.5
**HoLEP**	21.5 ± 8.5	10.1 ± 5.1	9.1 ± 5.1	8.6 ± 5.3	8.8 ± 4.5
**P value**	0.241	0.054	0.738	0.385	0.453
**IPSS voiding**					
**PVP**	12.1 ± 5.2	5.9 ± 5.0	4.4 ± 3.5	4.8 ± 3.9	5.2 ± 4.1
**HoLEP**	13.2 ± 5.4	3.8 ± 3.3	3.5 ± 3.2	4.3 ± 3.7	4.5 ± 3.5
**P value**	0.057	<0.001	0.103	0.529	0.409
**IPSS storage**					
**PVP**	8.3 ± 3.8	5.8 ± 3.4	5.1 ± 3.0	5.0 ± 3.4	4.5 ± 2.6
**HoLEP**	8.3 ± 4.4	6.3 ± 3.3	5.6 ± 3.2	4.3 ± 2.7	4.4 ± 3.0
**P value**	0.940	0.271	0.281	0.193	0.809
**QoL**					
**PVP**	4.1 ± 2.2	2.4 ± 1.5	2.1 ± 1.3	2.1 ± 1.4	2.0 ± 1.3
**HoLEP**	4.2 ± 1.1	2.5 ± 1.4	2.4 ± 1.4	2.4 ± 1.3	1.9 ± 1.3
**P value**	0.640	0.694	0.108	0.234	0.747

Values are mean s ± standard deviation. PVP, photoselective vaporization of prostate; HoLEP holmium laser enucleation of the prostate; IPSS, International Prostate Symptoms Score; QoL, quality of life

## Discussion

Prostate volume is a major factor in decisions regarding treatment for BOO by BPH. Several surgical strategies for BOO secondary to large and moderate prostate volumes can be used, including open prostatectomy, TURP, PVP, and HoLEP. However, urologic surgeons might hesitate to perform surgery in a patient with a small prostate volume due to the possibility of decreased procedural efficacy. Thus the treatment options for LUTS due to a small prostate volume comprise medical interventions, such asα adrenergic-receptor blockers [12]. However, medical treatment fails to make desirable outcomes in some patients, who therefore remains surgery is important option for treatment of small prostate volume.

New laser technologies, such as PVP using a GreenLight laser and HoLEP, for surgical treatment of BOO secondary to BPH have been adopted rapidly. In a meta-analysis comparing PVP or HoLEP with TURP, the two laser techniques exhibited promising efficacy and low intraoperative and early postoperative morbidity; e.g., reduced blood loss, significantly shorter catheterization duration, and shorter length of hospital stay [13–15]. However, few reports have compared PVP and HoLEP in patients with a small prostate volume, and high-quality evidence of comparing with the efficacy and safety of PVP and HoLEP in small prostate volumes is lacking. Therefore, we compared PVP with HoLEP for the treatment of BPH in terms of their effectiveness and incidence of intra- and postoperative complications, focusing especially on patients with a small prostate volume.

The precise cut-off value for a small prostate is unclear. A cut-off value of 40 mL for a small prostate volume in this study was based on previous reports [2, 3]. Kaplan and colleagues divided a cohort into three groups according to prostate volume, and reported that patients with BPH >40 mL volume tended to benefit from combination therapy with α adrenergic blocker and 5α reductase inhibitor compared with patients with a small prostate volume (< 25 mL). Thus prostate volume is related to the prognosis of patients with BPH [12]. In contrast, another study of 63 patients who underwent urodynamic study reported that prostate volume is related to BOO in patients with a prostate volume > 30 mL by TRUS. However, this relationship was not present in patients with prostate volume < 30 mL [16].

Elmansy et al. reported that PVP and HoLEP were effective for LUTS due to BPH with a >60 mL prostate volume in a randomized control trial that compared PVP using a 120W HPS system with HoLEP [17]. Furthermore, short-term subjective parameters (Qmax and PVP) may exhibit greater improvement following HoLEP compared to PVP. A retrospective study that compared Holmium:YAG transurethral incision (Hol TUIP)with PVP in patients with BOO secondary to a small prostate volume was performed in the same center [9]. Patients with prostates<40 mL were included in the study. Both surgical options were found to be effective and safe treatment modalities for small prostates, as evidenced by similar improvements in IPSS score, IPSS QoL score, Qmax, and PVR at the 60-month follow-up. However, the mean operation, hospitalization, and catheterization durations were superior in the Hol TUIP group.

To date only one retrospective study has assessed the efficacy of PVP and Hol TUIP for BPH in a small prostate [9]. Due to the lack of data comparing PVP with HoLEP in patients with a small prostate volume, we aimed to provide clinically relevant data. In this present study, there was no significant difference between the two groups concerning the preoperative parameters, with the exception of PVR. Improvement of subjective and objective parameters was comparable in both groups, with the exception of the voiding IPSS subscore at 1 month postoperatively. We assumed that early recovery of voiding symptoms in the HoLEP group was due to more-radical removal of prostate adenoma than in the PVP group. However, because the voiding IPSS subscore did not differ significantly at the 3, 6, and 12-month follow ups, and Qmax was comparable, PVP is not inferior to HoLEP. Furthermore, we found no difference between the groups in terms of complications. Bladder neck contracture after surgery is a frequent occurrence in prostates of small volume [18, 19]. In this study the PVP group (six cases, 3.4%) showed a tendency to a higher rate of BNC compared with the HoLEP group (two cases, 1.2%); however, the difference was not significant (P < 0.05). We suggested that bladder neck incisions at the 5 and 7 o’clock positions at the end of the operation in prostates of small volume may reduce the incidence of BNC [20]. Therefore, PVP and HoLEP are safe and effective minimally invasive treatment options for prostates of small volume with favorable results. This study highlights the advantages of PVP and HoLEP for treatment of LUTS secondary to BPH in patients of with a prostate of small volume.

Our study has a number of limitations. First, since the data were collected retrospectively despite use of prospectively maintained GreenLight and HoLEP data, this study might have been subject to selection bias. The second limitation was the lack of long-term follow up after surgery. Therefore, the efficacy and safety of PVP and HoLEP for prostates of small volume should be confirmed by prospective controlled studies including a greater number of patients and prolonged follow-up duration. However, our results are clinically meaningful, as few studies comparing PVP and HoLEP for prostates of small volume have been reported.

## Conclusions

This study demonstrates that PVP and HoLEP are safe and effective for treatment of patients with BOO due to a small prostate volume. PVP and HoLEP should thus considered feasible alternatives to TURP in patients with a small prostate volume.
